# Serum protein electrophoresis pattern in patients living with HIV: frequency of possible abnormalities in Iranian patients

**Published:** 2019-10

**Authors:** Zohreh Nozarian, Vahid Mehrtash, Alireza Abdollahi, Saloomeh Aeinehsazi, Atieh Khorsand, Arezoo Eftekhar-Javadi, Masoumeh Safaei, Fatemeh Nili

**Affiliations:** 1 Department of Pathology, School of Medicine, Imam Hospital Complex, Tehran University of Medical Sciences, Tehran, Iran; 2 Department of Pathology, School of Medicine, Sina Hospital, Tehran University of Medical Sciences, Tehran, Iran

**Keywords:** Electrophoresis, Polyclonal gammopathy, Human immunodeficiency virus, Proteins, Analytes

## Abstract

**Background and Objectives:**

This prospective case-control study was conducted to evaluate abnormal serum protein electrophoresis (SPEP) patterns in patients living with human immunodeficiency virus (HIV) and its relation with disease severity markers and anti-retroviral treatment status.

**Materials and Methods:**

Thirty-seven HIV-positive patients and 24 healthy individuals were evaluated in the course of this study. The healthy HIV-negative individuals were selected as control group. Pregnant women, patients with malignancies, children, hepatitis B- and/or C-positive patients, those with a history of an autoimmune disease, or previous corticosteroid administration were excluded. SPEP—which detects serum levels of albumin, total protein, gammaglobulin—, CD4+ T-cell counts, viral load, and antiretroviral treatment status were assessed. Data were analyzed by SPSS™ software.

**Results:**

Twelve patients (32 percent) demonstrated polyclonal gammopathy on SPEP, while only 1 (4 percent) healthy individual had the same pattern (P-value = 0.007). No statistically significant connection between SPEP patterns and antiretroviral treatment status was observed (P-value > 0.05). Interestingly no statistically significant relationship between CD4+ T-cell counts and polyclonal gammopathy was discerned. No statistically significant difference was observed between the two groups with regards to serum albumin and total protein levels. The serum albumin to total protein percentage, serum gamma globulin to total protein percentage, and serum albumin to globulin ratio was compared between the groups and a statistically significant difference was observed.

**Conclusion:**

Polyclonal gammopathy on SPEP is common among HIV-infected patients. Moreover, the SPEP patterns cannot be used as an indication of a patient’s negative or positive response to treatment.

## INTRODUCTION

The quintessential specification of HIV infection is a decrease in CD4+ T-cell levels accompanied by chronic hyperactivation in adaptive and innate immune systems (1, 2). This decline in CD4+ T-cell levels is caused by the chronic hyperactivation in part, which itself is a clear indication of the disease’s progress (3, 4).

In addition to T-cells, B-cells are also affected by HIV infection. This effect is manifested by changes in their subpopulation, their unusual hyperactivation—which can be discerned by an increase in surface activation markers, polyclonal activation, higher number of cell turnovers, and an increased ratio of plasmablasts to B-cells—and also increased levels of immunoglobulins (5–9). As a consequence of the increase in HIV viral load, B-cells produce more immunoglobulins (which can be pointed at HIV epitopes). These changes can be detected in serum protein electrophoresis (SPEP) as monoclonal and polyclonal bands (10, 11). In spite of the increase of serum immunoglobulins in HIV-positive patients, the T-cell-dependent and -independent antigen-specific antibody formation impairment is observed (12, 13). HIV-positive patients are at a higher risk for plasma cell disorders, ranging from polyclonal hypergammaglobulinemia and monoclonal gammopathy to symptomatic multiple myeloma (14).

Previous studies on patients who have undergone highly active antiretroviral therapy (HAART) have shown that despite commensurate CD4+ T-cell restoration, traces of residual inflammation or increased immune activation still remains (1). In fact, monoclonal gammopathy was treated in only half the patients who had received HAART (15). In the present study, the aim is to determine the prevalence of hypergammaglobulinemia in the HIV-positive patients in different stages of the disease, and the way in which HAART can influence B-cell dysregulation restoration. The proposed hypothesis is that SPEP patterns are abnormal in HIV-positive patients who have not undergone treatment and correlate with markers of disease severity.

## MATERIALS AND METHODS

This case-control study was prospectively performed in Imam Khomeini Hospital, Tehran University of Medical Sciences, Tehran, Iran. The case group comprised of all HIV-positive patients referred to Iranian research center for HIV/AIDS (IRCHA) during spring and summer of 2016. They had already been diagnosed using enzyme-linked immunosorbent assay (ELISA) and confirmed to be HIV-positive by HIV Western Blot method according to The Centers for Disease Control and Prevention (CDC) guideline (16). The control group consisted of healthy HIV-negative individuals referred for annual checkups to clinical laboratory of Valiasr hospital, an affiliated laboratory of Imam Khomeini hospital. We excluded pregnant women, patients with malignancies, children (≤ 12 years old), hepatitis B- and/or C-positive patients, those with a history of an auto-immune disease, or previous corticosteroid administration. The study was accomplished in accord to the Declaration of Helsinki guidelines. SPEP was performed on serum proteins for both HIV-positive patients and healthy individuals using a CAPILLARYS 2 Flex Piercing capillaryelectrophoresis system according to manufacturer’s instruction (Sebia, Lisses, France). Flow cytometry was performed to count CD4+ T-cells on EDTA-anticoagulated blood using the PAS flow cytometer according to manufacturer’s instruction (Partec GmbH, Münster • Germany). Also, the HIV viral load was measured by PCR.

Data were analyzed by applying t-test and chi-squared test to compare case group with control group using the SPSS, version 19.0 software. Study results are statistically significant if the p-values of the data analyses are less than 0.05.

## RESULTS

Thirty-seven HIV-positive patients as case group and 24 healthy individuals as control group met our inclusion criteria and were evaluated in the course of this study.

Among the patients, there were 4 women (10.8 percent) and 33 men (89.2 percent). In the control group, gender distribution was as follows: 8 (33 percent) women versus 16 (67 percent) men. The mean age of control group was 39.62 years and the mean age of case group was 40.73.

**Fig. 1:**
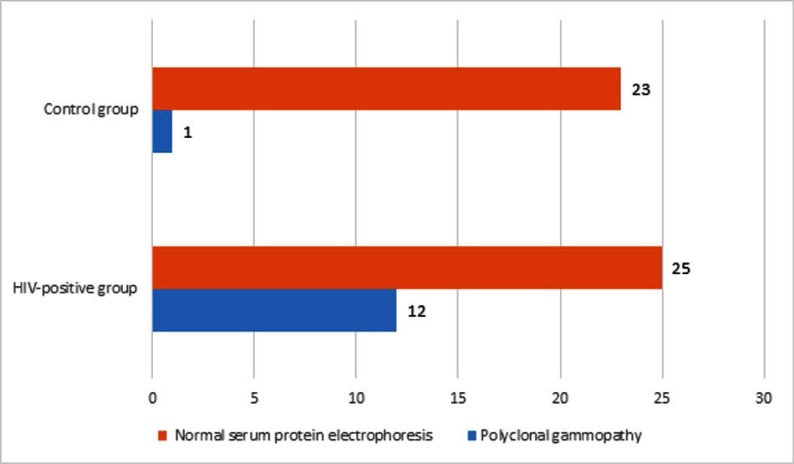
Serum protein electrophoresis pattern among HIV-positive group and control group (P-value = 0.007).

Twelve patients (32 percent) demonstrated polyclonal gammopathy on SPEP, while only 1 (4 percent) healthy individual had the same pattern (Fig. 1). These results show a statistically significant difference between the two groups (P-value = 0.007). The rest of the study group had a normal SPEP pattern; 68 percent in patients and 96 percent in healthy individuals.

No abnormal SPEP pattern was found among the healthy women, and only 1 out of the 16 healthy men (6 percent) demonstrated polyclonal gammopathy. Of the 4 female patients, 2 (50 percent) had normal SPEP pattern and 2 (50 percent) had polyclonal gammopathy (P-value = 0.5). Of the 33 male patients, 10 (30 percent) showed polyclonal gammopathy and the rest (70 percent) had normal SPEP pattern (P-value = 0.6).

Comparing HIV-negative and HIV-positive women in terms of presence of polyclonal gammopathy on SPEP, a statistically significant difference between them was observed (P-value = 0.02). On the other hand, no statistically significant difference was observed when healthy men and male patients were compared (P-value = 0.1).

The study population was divided into three age groups of 20 to 40-year-olds, 40 to 60-year-olds, and those over 60, and SPEP patterns was compared between healthy individuals and patients in these age groups. In the 20–40 age group, 4 out of 14 patients (36.4 percent) and 1 out of 14 healthy individuals (7.1 percent) demonstrated a polyclonal gammopathy. In the 40–60 age group, 7 out of 24 patients (29.2 percent) and none of the 7 healthy individuals demonstrated a polyclonal gammopathy pattern. In the over 60 age group, only 1 out of 2 patients (50 percent) and none of the healthy individuals demonstrated a polyclonal gammopathy. Given the greater than 0.05 P-value, no statistically significant difference was observed between these two groups.

**Fig. 2:**
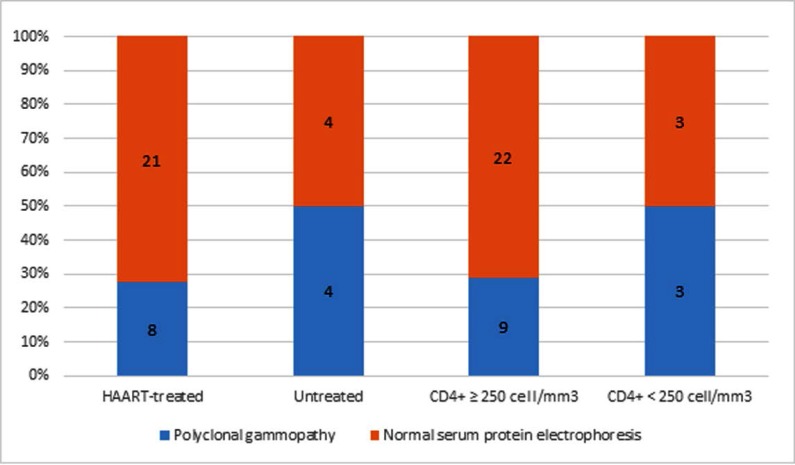
Comparison of serum protein electrophoresis pattern among HIV-positive patients according to CD4+ cell count and antiretroviral therapy condition, both of which had p-values greater than 0.05. HAART: highly active antiretroviral therapy

Relations between CD4+ T-cell level and status of antiretroviral treatment with SPEP patterns was evaluated (Fig. 2). Eight patients had not received antiretroviral treatment. Among these, 4 (50 percent) demonstrated a polyclonal gammopathy. Twenty-nine had received antiretroviral treatment among whom 8 (28 percent) demonstrated a polyclonal gammopathy. No statistically significant connection between SPEP patterns and status of antiretroviral treatment in patients was observed (P-value > 0.05). On the other hand, when recipients of antiretroviral treatment were compared with healthy individuals among whom 1 out of 24 (4 percent) showed a polyclonal gammopathy pattern, a statistically significant difference was observed (P-value < 0.02).

In a comparison between HIV-positive patients who had not undergone antiretroviral therapy and healthy individuals, polyclonal gammopathy levels showed a statistically significant difference (P-value < 0.002).

Six of the patients had a CD4+ T-cell count of less than 250/ml, and 3 of these (50 percent) demonstrated polyclonal gammopathy. The CD4+ T-cell count was greater than 250/ml in 31 patients, and 9 of these (29 percent) demonstrated polyclonal gammopathy. No statistically significant relationship between CD4+ T-cell counts and presence of polyclonal gammopathy was observed in the present study (P-value > 0.05).

One (4 percent) of the healthy individuals demonstrated polyclonal gammopathy. Compared to patients with a CD4+ T-cell count less than 250/ml, a statistically significant difference was observed in terms of SPEP patterns (P-value = 0.02). Furthermore, compared to patients with a CD4+ T-cell count > 250/ml, another statistically significant difference can be seen in this regard (P-value = 0.002). Since the viral load was unidentifiable among all patients, examining its connection with SPEP patterns was not possible in this study.

Concentrations of total protein and albumin, albumin to total protein percentage (A/TP), gamma globulin to total protein percentage (G/TP), and albumin to globulin (A: G) ratio in the two groups were calculated (Table 1).

**Table 1:** Concentrations of total protein, albumin, A/TP, G/TP, A:G in the two groups

	**HIV positive (n = 37)**	**Control (= 24)**	**P-value**
Total protein g/dL, mean (95% CI)	7.72 (7.45–8)	7.8 (7.57–8.02)	0.6
Albumin g/dL, mean (95% CI)	4.4 (4.27–4.54)	4.59 (4.25–4.93)	0.2
A/TP, mean (95% CI)	57.29% (55.7%–59%)	60.57% (58.9%–62%)	0.005
G/TP, mean (95% CI)	18.96% (17.3%–20.6%)	15.08% (13.91%–17.28%)	0.001
A:G, mean (95% CI)	1.35 (1.3–1.43)	1.55 (1.45–1.65)	0.002

HIV: Human immunodeficiency virus, A/TP: Albumin to total protein percentage, G/TP: Globulin to total protein percentage, A:G: Albumin to globulin ratio

## DISCUSSION

In the present study, we demonstrated that 32 percent of HIV-positive patients and 4 percent of healthy individuals showed a polyclonal gammopathy, pointing to a statistically significant difference between the two groups (P-value = 0.007). Among the HIV-positive patients, 29 (78 percent) had received antiretroviral treatment, while the other 8 (22 percent) had not received antiretroviral treatment. Four (50 percent) of those who had not received antiretroviral treatment showed a polyclonal gammopathy, whereas 8 (29 percent) of the recipients of antiretroviral treatment showed a polyclonal gammopathy. No statistically significant connection between antiretroviral treatment status and the SPEP patterns was observed. On the other hand, a statistically significant difference was observed between HIV-positive patients who had received antiretroviral treatment and the control group with regards to SPEP patterns (P-value < 0.02). Also, when patients who had not received antiretroviral treatment were compared to healthy individuals, a statistically significant difference was observed (P-value = 0.002).

In their study, Zemlin et al. (17) compared 70 HIV-positive patients who had undergone antiretroviral treatment with 42 HIV-negative individuals in a control group and showed that the SPEP had a polyclonal gammopathy pattern in 44 percent of the patients and 10 percent of the healthy individuals (P-value < 0.01). The present study also showed a statistically significant difference in the SPEP patterns between HIV-positive patients who have not undergone treatment and healthy individuals.

In the present study, comparison of SPEP patterns between healthy individuals and patients with CD4+ levels of higher and lower than 250/ml where P-values were 0.02 and 0.002, respectively, show a statistically significant difference. In patients with lower levels of CD4+, this difference was more conspicuous. Regarding the connection between viral load and SPEP patterns, we could not come to a concrete conclusion, because our study group had an immeasurable viral load. Similarly, in the Zemlin study the CD4+ T-cell serum levels among individuals with hypergammaglobulinemia or abnormal SPEP patterns were lower in a statistically significant way, but no statistically significant difference between viral load and SPEP patterns could be discerned.

In the current study, albumin and total protein levels were lower among the patients compared to the control group but in both situations no statistical significance was reached (P-values of 0.2 and 0.6, respectively). In the Zelmin study on the other hand, while the albumin levels were lower in patients (P-value = 0.3), total serum protein levels were higher in them (P-value = 0.08). Again, Zelmin et al. failed to show any statistical significance in this regard. The present study also shows that the ratio of serum albumin to total plasma protein in patients is lower compared to the control group in a statistically significant way (P-value = 0.005).

In another study, Adedeji et al. (18) chose a group of HIV-1-infected individuals who had not developed AIDS despite not receiving antiretroviral treatment, and compared them to another group of patients who had undergone HAART. SPEP abnormalities indicative of chronic inflammation were significantly higher in patients who had not received the HAART (P-value = 0.001). In the present study, polyclonal gammopathy pattern was also more common among the patients who had not undergone HAART compared to those who had, but the difference was not statistically significant (P-value > 0.05).

Konstantinopoulos et al. (10) examined the SPEP patterns and immunoglobin levels in 230 HIV-1 patients and carried out immunofixation on samples with abnormal SPEP. SPEP pattern was normal in 83.8 percent of the samples, 8.1 percent of samples showed changes as oligoclonal bands, and there was low concentration (less than 5 percent of the whole proteins) monoclonal band in 4.4 percent of samples. Both hypogammaglobulinemia and polyclonal hypergammaglobulinemia were observed in 1.9 percent of the study population. Whereas in our study, among HIV-positive patients, we observed only polyclonal gammopathy in a much larger quantity (32 percent) and none of the patients showed monoclonal gammopathy.

Another study that shows monoclonal and oligoclonalgammopathy among HIV-positive patients in contrast to our study was conducted by Van Vuuren et al. (19) They studied 368 patients who were undergoing HAART. The monoclonal band was observed in 12 patients (3.2 percent), while 14 patients (3.8 percent) had an oligoclonal band. That study showed that compared to a normal population, monoclonal and oligoclonal bands are more common among HIV-patients undergoing treatment. No patients with monoclonal bands were observed in the present study, though.

Sarro et al. (20) studied the role of SPEP in monitoring the progress of disease in HIV-positive patients on antiretroviral treatment. In total, 220 subjects were examined in that study which were divided into four groups: symptomatic HIV-positive patients who were undergoing treatment, symptomatic HIV-positive patients who were not undergoing treatment, asymptomatic HIV-positive patients, and healthy blood donors. The SPEP results on these groups showed that gamma globulin density was lowest among the healthy individuals, medium among symptomatic and asymptomatic HIV-positive patients who were undergoing antiretroviral treatment, and highest among symptomatic HIV-positive patients who were not undergoing antiretroviral treatment. The Sarro study shows that there is a statistically significant connection between gamma globulin density and the disease stage (P-value < 0.001). These findings are in line with the results of the present study where polyclonal gammopathy patterns were most common among untreated patients, then among treated patients, and least common among healthy individuals.

In their study, Casanova et al. (21) screened 81 volunteers for HIV infection, 30 of whom were HIV-positive and the other 51 were healthy. In that study, 60 percent of HIV-positive patients had hyperproteinemia, and all of them had hypoalbuminemia and hypergammaglobulinemia according to their CD4+ T-cell counts. In the present study, however, no hypoalbuminemia was observed in HIV-positive patients, but the mean albumin levels among the patients were lower compared to healthy individuals. Furthermore, only 32 percent of the patients who participated in the present study had hypergammaglobulinemia, and the total protein mean was lower in the patients compared to the control group. These results contradict the findings of the Casanova et al. study completely.

Soong and Riley (22) assessed the nutritional status of HIV-positive patients by measuring their serum total protein and albumin levels. Their study showed that total protein is significantly higher in HIV-positive patients (7.43 ± 0.13 g%) compared to their control group (7.07 ± 0.22 g%), whereas the present study led to contradictory results; total protein mean was 7.72 ± 0.8 g% among the HIV-positive patients and 7.80 ± 0.5 g% in the control group. This finding shows lower serum albumin levels in HIV-positive patients compared to healthy individuals. In the Patil study, serum albumin level mean was 2.67 ± 0.34 g% among the HIV-positive patients and 4.45 ± 0.26 g% among the healthy individuals. In the present study, serum albumin level means for HIV-positive patients and healthy individuals were 4.40 ± 0.40 g% and 4.59 ± 0.80 g%, respectively. The findings of these two studies show that serum albumin levels were lower in HIV-positive patients. In the Patil study, A:G ratio mean in the control group was 1.70 ± 0.26 g%, while this ratio among the HIV-positive patients was down to 0.56 ± 0.10 g%. In the present study, on the other hand, A:G ratio was 1.50 ± 0.20 g% in the control group and 1.35 ± 0.20 g% in HIV-positive patients. As such, the results of this study are in contrast of the findings of the Patil study. Given the limited scope of that study, as well as the present one—where factors such as nutritional and socio-economic status, HAART regimen, provided care for infection and malignancy management were not considered—enough evidence to draw a firm conclusion was not on hand. Since the administrated HAART regimen is generally determined in a percase fashion, comparing the present study to those of our peers in terms of HAART regimen was not possible. It should be noted that in many of the studies on SPEP patterns in HIV-positive patients, monoclonal gammopathy and its changes during the development of AIDS, treatment and progression towards malignancy have been examined, whereas, due to the limited size of the study population, monoclonal gammopathy was not taken into account in the present study.

According to the findings of our research, a statistically significant difference in the SPEP patterns was observed between female patients and healthy women, but since the study population was not large enough, further studies must be conducted before a firm conclusion can be made.

Serum albumin to total protein percentage, serum gamma globulin levels and A:G ratio were lower than normal in the patients which could be due to the progression of the disease or irregularities in their nutrition. On the other hand, no statistically significant difference could be observed in serum albumin and total protein levels between patients and controls which could be because of the serum albumin levels calculation method used—based on its percentage of the total protein according to densitometry method rather than calorimetry method.

One of the possible clinical applications of the present study which could prove useful is the fact that SPEP pattern can be utilized in the assessment of patients’ response to antiretroviral therapy by prediction of CD4+ count, and viral load status. Regrettably, although SPEP differences were observed between patients with CD4+ levels of 250/ml/ml and higher, and those with CD4+ levels of lower than 250/ml/ml, the connection was not statistically significant. Furthermore, considering the fact that the viral load was not measured in all HIV-positive patients who were part of this study, SPEP pattern could not be proven to be a reliable criterion for the prediction of viral load status. To confirm the abovementioned connection, studies with larger statistical populations are recommended.

Given the statistically significant difference in SPEP patterns between HIV-positive patients and healthy individuals, which indicates gammopathy in HIV-positive patients, and a number of previous studies showing higher prevalence of hematologic malignancies in patients with abnormal SPEP patterns (23, 24), clinicians should take the higher risk of malignancy into account when dealing with HIV-positive patients who present with abnormal SPEP patterns. Accordingly, HIV-positive patients might suffer from multiple myeloma at a much younger age compared to general population (25). Furthermore, these patients demonstrate worse prognosis and shorter life spans. Additionally, multiple myeloma is an indication of initiating HAART in addition to chemotherapy (14).

In conclusion, polyclonal gammopathy on SPEP is common among HIV-positive patients which is a determination of B-cell hyperactivation. While treatment improves CD4+ level, it has no impact on SPEP pattern; therefore, SPEP cannot be used as indication of the patient’s response to treatment.
